# Using ChatGPT for language editing in scientific articles

**DOI:** 10.1186/s40902-023-00381-x

**Published:** 2023-03-08

**Authors:** Seong-Gon Kim

**Affiliations:** Department of Oral and Maxillofacial Surgery, College of Dentistry, Gangneung-Wonju National University, Gangneung, 28644 Republic of Korea

In recent times, there has been a lot of buzz surrounding the artificial intelligence (AI) chatbot. Some articles have even listed the chatbot as an author [1, 2]. However, authorship is an ethical issue of significant importance in scientific articles, and it has become a critical matter in scientific journals. A recent publication in Nature [3] stated that an AI chatbot cannot be listed as an author of a scientific article since it cannot take responsibility for the article’s claims. One of the most renowned AI-powered chatbots is ChatGPT, developed by OpenAI in San Francisco, California, USA.

As ChatGPT is freeware until now, it is available for anybody [4]. I submitted several questions to ChatGPT and asked replies. My question was “When streptozotocin-induced diabetes is prepared in growing rats, can you predict its effect on the facial bone growth pattern?” And it replied that “Streptozotocin-induced diabetes can have systemic effects on the body, including alterations in bone metabolism and growth. In growing rats, diabetes induced by streptozotocin can lead to impaired bone formation and remodeling, resulting in decreased bone volume and mineral density. These changes can affect the growth pattern of the facial bones, leading to alterations in the size and shape of the maxilla and mandible. Additionally, diabetes can lead to delayed tooth eruption and impaired dental development, which can further impact the growth pattern of the facial bones. Therefore, it is possible that streptozotocin-induced diabetes in growing rats can have an effect on the facial bone growth pattern. However, the specific changes that occur would depend on the severity and duration of the diabetes, as well as other individual factors.” And I asked again that “Is there any reference for this topic?” Then, it replied that “Yes, there are many references on the effects of streptozotocin-induced diabetes on bone growth patterns in rats. Here are a few examples:

1. Yamamoto M, Kawashima N, Nishimura N, Konishi T, Udagawa N. Streptozotocin-induced diabetes mellitus suppresses mandibular condylar growth in rats. J Bone Miner Metab. 2005;23(2):135-141.
2. Luder HU, Gratz KW, Schroeder HE. Effects of streptozotocin-induced diabetes on alveolar bone remodeling in growing rats. J Periodontol. 1991;62(3):177-183.
3. Sisman Y, Ercan E, Soylu E, et al. Effects of streptozotocin-induced diabetes mellitus on orthodontic tooth movement and associated root resorption in rats. Eur J Orthod. 2014;36(6):689-695.
4. Guncu GN, Akman S, Yavuz T, Cetinkaya BO, Toker H. Effects of streptozotocin-induced diabetes mellitus on mandibular condylar cartilage in rats: a histomorphometric study. Arch Oral Biol. 2007;52(10):946-952.

These studies and others suggest that streptozotocin-induced diabetes can have a negative impact on bone growth and development in rats, including the facial bones.” I searched whether these references are real or fake. Unfortunately, all references are fake including the fake authors. However, its other performance such as an editing English grammar was wonderful.

Many authors from non-English speaking countries have utilized the language editing service provided by various publishers. In comparison to paid English-editing service, ChatGPT has shown to be more advantageous in some aspects. Its ability to produce more refined sentences surpasses those of the unedited ones. It is capable of providing an edited paragraph within seconds and, currently, it is available for free to everyone.

The primary objective of a scientific article is to convey new information that is supported by evidence, and all claims made by the authors should be thoroughly scrutinized before being published. While authors may seek assistance from English-editing services during the publication process, such services should not be listed as co-authors. If ChatGPT is only used for language editing purposes, then there is no issue with using it to prepare scientific articles. However, any new ideas generated by ChatGPT should be validated through actual experiments and their results should be verified by humans. As demonstrated earlier, the current version of ChatGPT has the potential to generate false information, so it is important for human authors to carefully review and validate the information generated by ChatGPT before including it in their articles. Additionally, any assistance provided by AI should be disclosed in the article [5].

## Data Availability

Not applicable.
